# Are the facial gender and facial age variants of the composite face illusion products of a common mechanism?

**DOI:** 10.3758/s13423-019-01684-9

**Published:** 2019-12-10

**Authors:** Katie L. H. Gray, Yvonne Guillemin, Zarus Cenac, Sophie Gibbons, Tim Vestner, Richard Cook

**Affiliations:** 1 School of Psychology and Clinical Language Sciences, University of Reading, Reading, UK; 2 Department of Psychology, City, University of London, London, UK; 3 Department of Psychological Sciences, Birkbeck, University of London, London, UK

**Keywords:** Composite face illusion, Facial gender, Facial age, Individual differences, Psychophysics

## Abstract

When the upper half of one face (‘target region’) is spatially aligned with the lower half of another (‘distractor region’), the two halves appear to fuse together perceptually, changing observers’ subjective perception of the target region. This ‘composite face illusion’ is regarded as a key hallmark of holistic face processing. Importantly, distractor regions bias observers’ subjective perception of target regions in systematic, predictable ways. For example, male and female distractor regions make target regions appear masculine and feminine; young and old distractor regions make target regions appear younger and older. In the present study, we first describe a novel psychophysical paradigm that yields precise reliable estimates of these perceptual biases. Next, we use this novel procedure to establish a clear relationship between observers’ susceptibility to the age and gender biases induced by the composite face illusion. This relationship is seen in a lab-based sample (*N* = 100) and is replicated in an independent sample tested online (*N* = 121). Our findings suggest that age and gender variants of the composite illusion may be different measures of a common structural binding process, with an origin early in the face-processing stream.

**Electronic supplementary material:**

The online version of this article (10.3758/s13423-019-01684-9) contains supplementary material, which is available to authorized users.

Upright faces are thought to engage holistic processing whereby local features are integrated into a unified whole for the purposes of accurate and efficient interpretation (Farah, Wilson, Drain, & Tanaka, 1998; McKone & Yovel, 2009; Piepers & Robbins, 2013). In the absence of canonical first-order facial information (e.g., when judging inverted or feature-scrambled faces), observers may be forced to base perceptual decisions on a piecemeal analysis of local features. The extent to which individuals process faces holistically is thought to determine their ability to recognize and interpret faces (DeGutis, Cohan, & Nakayama, 2014; Richler, Cheung, & Gauthier, 2011).

The composite face illusion is a key hallmark of holistic face processing. When the upper half of one face (the ‘target region’) is spatially aligned with the lower half of another (a task-irrelevant ‘distractor region’), the two halves appear to fuse together perceptually, changing observers’ subjective perception of the target region (Young, Hellawell, & Hay, 1987). The perceptual fusion observed is greatly reduced where one half is offset horizontally (‘misaligned’) or where aligned arrangements are presented upside down. This feature of the illusion suggests that the human visual system integrates distal facial information only in the presence of an intact faciotopy (Murphy, Gray, & Cook, 2017; Rossion, 2013).

The composite face illusion is not merely an interference effect where distractor regions hinder perceptual decisions about a target. Importantly, distractor regions bias observers’ subjective perception of target regions in systematic, predictable ways (Rossion, 2013). For example, male and female distractor regions make target regions appear masculine and feminine (Baudouin & Humphreys, 2006); young and old distractor regions make target regions appear younger and older (Hole & George, 2011); and happy and unhappy distractor regions make target regions seem happier and sorrowful (Calder, Young, Keane, & Dean, 2000).

It remains uncertain whether the different perceptual biases induced by composite face arrangements are different measures of a common structural binding process, or whether these biases should be thought of as independent illusory effects. Leading theoretical models posit that observers form a structural representation early in the face-processing stream (Bruce & Young, 1986; Haxby, Hoffman, & Gobbini, 2000), which forms a common basis for judgements about various facial attributes (e.g., identity, expression, age, gender). This initial modeling of face structure may generate the different biases induced by the composite face illusion. Alternatively, different perceptual biases may be products of attribute-specific holistic processing that occurs later in the face-processing stream—for example, the holistic processing of facial age (Hole & George, 2011) or facial gender (DeGutis, Chatterjee, Mercado, & Nakayama, 2012), per se.

We sought to distinguish these possibilities by comparing individuals’ susceptibility to the age and gender biases induced by the composite face illusion. If these biases are different measures of the same structural binding process, individuals’ susceptibility to one bias should predict their susceptibility to the other. However, if these illusory biases arise from attribute-specific holistic processing, we might expect little or no association between observers’ susceptibility to these biases.

## Measuring the composite face illusion using psychophysics

**Fig. 1:**
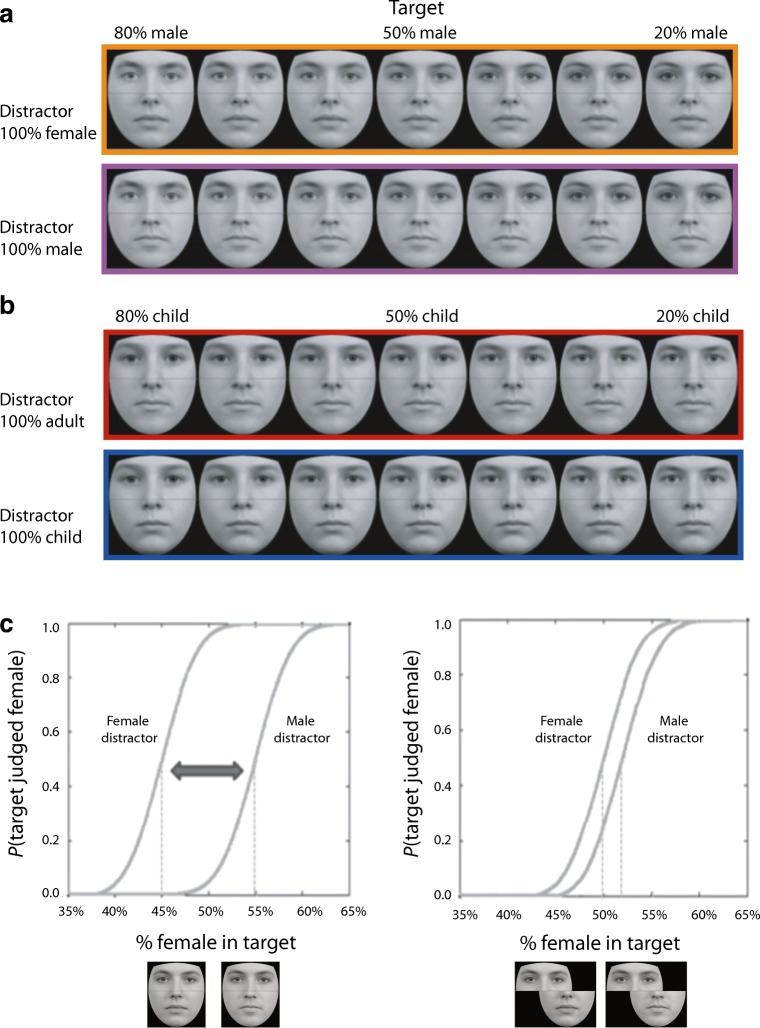
Stimuli used in the gender (**a**) and age (**b**) variants of the task. When shown upright and aligned, male and female distractors make target regions appear more masculine and feminine; child and adult distractors make target regions appear younger and older, respectively. Modulation of observers’ perception is inferred from shifts in their psychometric functions (**c**)

We measured individuals’ susceptibility to the age and gender biases induced by the composite face illusion using a novel psychophysical procedure (see Fig. 1). In our paradigm, the manifestation of the illusion is inferred from shifts in observers’ psychometric functions. Examining how different viewing conditions modulate psychometric functions has helped vision scientists document the behavior of other illusions in this field—notably, facial aftereffects (Leopold, O’Toole, Vetter, & Blanz, 2001; Webster & MacLeod, 2011). This approach also offers several advantages to those seeking to study the composite face illusion. In particular, researchers can dissociate the extent to which a distractor biases observers’ perception in a particular direction—the critical measure of the composite face illusion—from observers’ ability to detect and interpret the physical differences between target regions (i.e., the amount of internal noise associated with judgements about target regions). Crucially, the modeling of psychometric functions yields separate estimates of these independent parameters—the point of subjective equality (PSE) and function slope, respectively.

In both variants of our task, observers were asked to make binary categorization judgements about upper face halves (encompassing the eyes; target regions) drawn from morph continua, while ignoring lower face halves (encompassing the mouth; distractor regions) that were not subject to morphing (i.e., presented at 100% intensity). In the gender variant of the task, target regions were drawn from a continuum, blending an average male face with an average female face. Observers judged whether target regions depicted a male or female face, either in the presence of a distractor region cropped from the average male face or a distractor region cropped from the average female face. In the age variant of the task, target regions were drawn from a continuum blending an average child face with an average adult face. Observers judged whether target regions depicted a child or adult face, either in the presence of a distractor region cropped from the average child face or a distractor region cropped from the average adult face.

In both variants, participants’ responses were used to construct psychometric functions that modeled the relationship between response probability and the strength of the signal present in the target region (% female or % adult). Under our psychophysical approach, observers’ susceptibility to the illusion is inferred from the extent to which their psychometric functions diverge in different distractor conditions, inferred from the difference in PSE. To date, it has proved extremely difficult to measure individuals’ susceptibility to the composite face illusion in a reliable way (Richler & Gauthier, 2014). Importantly, however, estimates of test–retest reliability obtained using our psychophysical paradigm range from *r* = .602 to *r* = .775 (see Supplementary Material).

## Replicating the normative properties of the illusion

As described above, the composite face illusion manifests disproportionately when distractor and target regions are spatially aligned, and arrangements are presented upright. Little, if any, illusory distortion is seen when target and distractor regions are misaligned, or when aligned arrangements are presented upside down (Murphy et al., 2017; Rossion, 2013). Although the focus of the present paper is inter-observer variability in composite illusion susceptibility, we first sought to confirm that our psychophysical paradigm replicated these key features of the illusion.

Two samples of typical observers completed the gender (*N* = 19, *M*_age_ = 26 years, *SD*_age_ = 7.32 years, five males) and age (*N* = 19, *M*_age_ = 25 years, *SD*_age_ = 2.11 years, seven males) variants of our task. All participants had normal or corrected-to-normal vision and were tested in person, under controlled lab conditions. For all experiments described, ethical clearance was granted by the local ethics committee, and the studies were conducted in line with the ethical guidelines laid down in the Sixth (2008) Declaration of Helsinki.

### Materials and procedure

The average male and female faces, and the average child and adult faces used to construct the morph continua, were composites of eight faces sourced from the Radboud Face Database (Langner et al., 2010). Each continuum comprised seven levels that varied stimulus intensity from 20% to 80% in increments of 10%. Morphing was achieved through Morpheus Photo Morpher, Version 3.11 (Morpheus Software, Inc). Facial composites subtended ~6° vertically when viewed at 58 cm. In the misaligned condition, target and distractor halves were offset horizontally by ~3°. A thin grey line (~4 pixels) was inserted in between the target and distractor to help participants distinguish the to-be-judged regions (Rossion & Retter, 2015).

Each trial began with a fixation point, followed by a composite arrangement presented for 1,200 ms. Participants registered their categorization decision with a key-press response. Participants judged the target under three configuration conditions: aligned distractors within an upright arrangement, misaligned distractors within an upright arrangement, and aligned distractors within an inverted arrangement. For each condition, we constructed a psychometric function from 140 categorization decisions (7 target levels × 20 presentations). In total, observers therefore completed 840 trials (140 trials × 3 configuration conditions × 2 levels of distractor). Experimental programs were written in MATLAB (The MathWorks, Inc.) using Psychtoolbox (Brainard, 1997; Pelli, 1997). Psychometric functions were modeled by fitting cumulative Gaussian functions using the Palamedes toolbox (Prins & Kingdom, 2018). Our measure of function slope was the reciprocal of the standard deviation of the symmetric Gaussian distribution underlying each cumulative Gaussian function. Goodness of fit was evaluated using *p*Dev statistics.

### Results and discussion

#### Gender variant

**Fig. 2:**
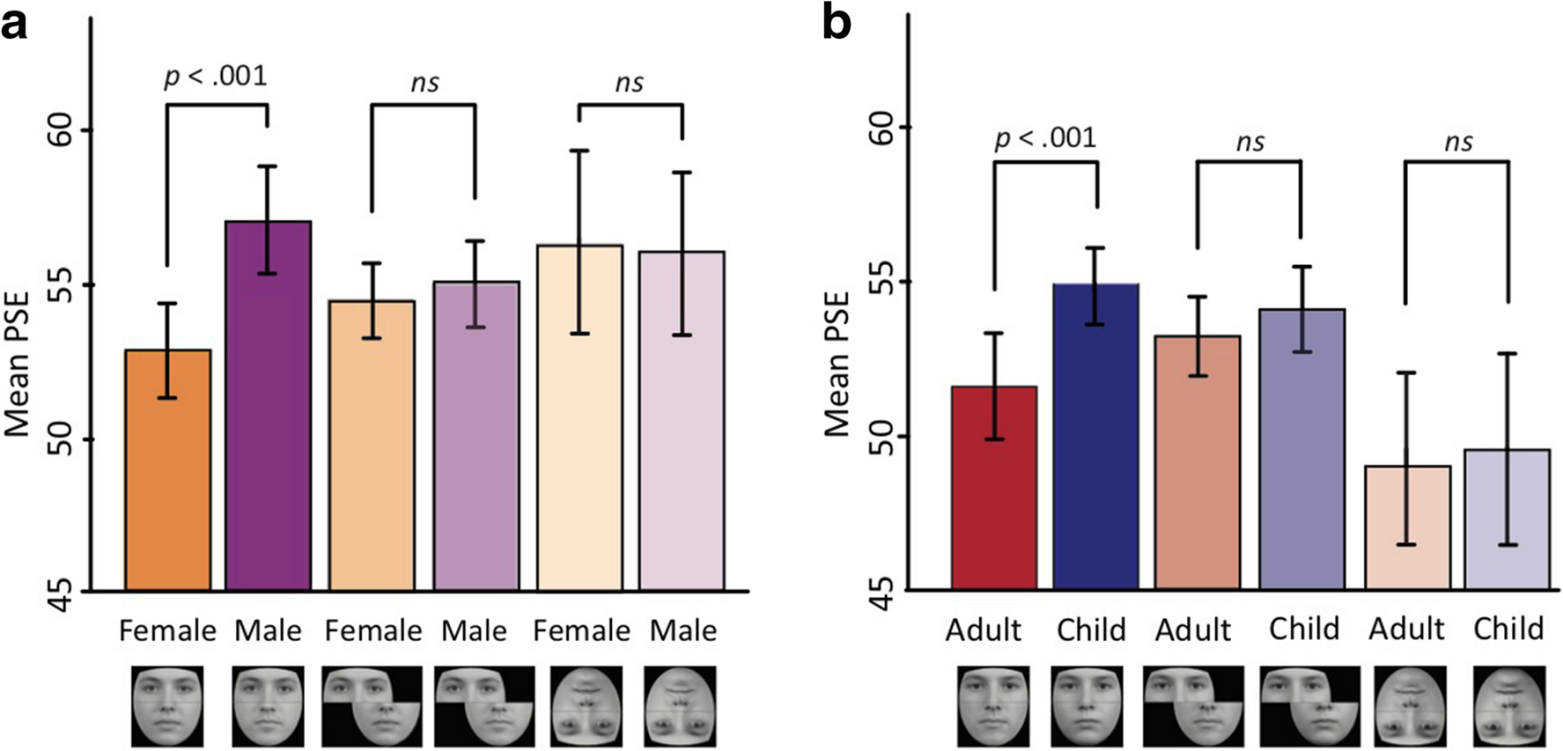
Results from two samples of typical observers (both *N*s = 19) who completed the gender (**a**) and age (**b**) variants of the task. Substantial shifts were seen when aligned composite arrangements were shown upright. Little or no modulation was seen when distractor regions were misaligned or when composite arrangements were shown upside down. Error bars denote ± *SEM.* PSE = point of subjective equality

The distribution of PSEs was analyzed using an ANOVA, with Distractor (female, male) and Configuration (aligned, misaligned, inverted) as within-subjects factors (see Fig. 2a). The analysis revealed a significant main effect of Distractor, *F*(1, 18) = 5.47, *p* = .031, η_p_^2^ = .23, a nonsignificant main effect of Configuration, *F*(2, 36) = .49, *p* = .62, η_p_^2^ = .03, and a significant Distractor × Configuration interaction, *F*(2, 36) = 5.78, *p* < .01, η_p_^2^ = .24. Planned comparisons revealed a significant PSE shift when composites were upright and aligned, *t*(18) = 3.12, *p* < .01. Observers were more likely to judge the target to be male-like in the presence of the male distractor (*M* = .57, *SD* =.07) than in the presence of the female distractor (*M* = .53, *SD* = .07). We failed to observe significant PSE shifts when the distractor regions were misaligned, *t*(18) = .85, *p* = .41, or inverted, *t*(18) = .29, *p* = .78.

#### Age variant

The distribution of PSEs was analyzed using an ANOVA, with Distractor (child, adult) and Configuration (aligned, misaligned, inverted) as within-subjects factors (see Fig. 2b). The analysis revealed a significant main effect of Distractor, *F*(1, 18) = 25.68, *p* < .001, η_p_^2^ = .59, a main effect of Configuration, *F*(2, 36) = 3.74, *p* = .03, η_p_^2^ = .17, and a significant Distractor × Configuration interaction, *F*(2, 36) = 3.63, *p* = .04; η_p_^2^ = .17. Planned comparisons revealed a significant PSE shift when composites were upright and aligned, *t*(18) = 4.14, *p* = .001. Observers were more likely to judge the target to be adult-like in the presence of the adult distractor (*M* = .56, *SD* =.06) than in the presence of the child distractor (*M* = .51, *SD* = .07). We observed nonsignificant PSE shifts when the distractor regions were misaligned, *t*(18) = 1.28, *p* = .22, or inverted, *t*(18) = .55, *p* = .59.

Consistent with the documented normative properties of the composite face illusion (Murphy et al., 2017; Rossion, 2013), our paradigm produced marked shifts only when distractors were spatially aligned and arrangements were presented upright; little or no modulation was seen when distractor regions were misaligned or when composite arrangements were shown upside down. This selective modulation indicates that PSE shifts observed in upright-aligned arrangements were attributable to the illusion, not to response bias.

## Comparing individuals’ susceptibility to the age and gender composite illusions

Next, we sought to examine whether individuals’ susceptibility to the age and gender composite illusions is related. One hundred typical observers (*M*_age_ = 23 years, *SD*_age_ = 4.84 years, 36 males) completed both the gender and age variants of our task. All participants were tested in person, under controlled lab conditions. Eight of these observers were replacements for participants for whom we were unable to model psychometric functions in one or more conditions (i.e., there was no systematic relationship between stimulus intensity and their pattern of responding).

We modeled eight functions for each observer. In addition to the four aligned conditions (aligned child distractor, aligned adult distractor, aligned male distractor, aligned female distractor), we also modeled four functions describing observers’ categorization decisions when distractor regions were misaligned (misaligned child distractor, misaligned adult distractor, misaligned male distractor, misaligned female distractor). Each psychometric function was estimated from 140 categorization decisions (7 target levels × 20 presentations). Over the course of two testing sessions, each observer completed 1,120 trials (140 trials × 2 levels of distractor × 2 alignment conditions × 2 variants). Half the participants completed the age variant first, and half completed the gender variant first.

### Results and discussion

#### Gender variant

**Fig. 3:**
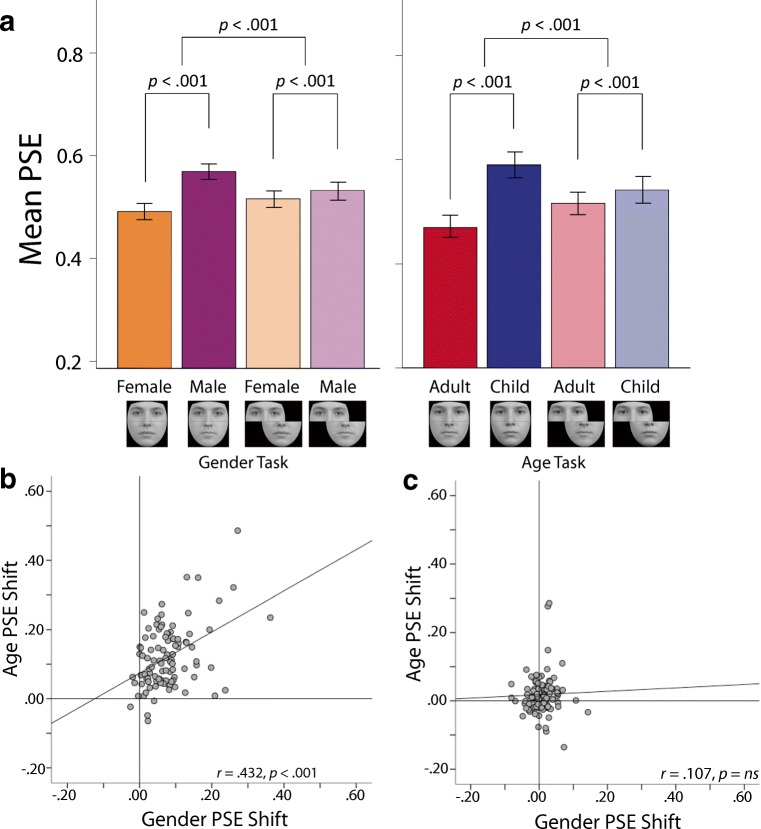
Results from the lab-based sample. As expected, substantial function shifts were induced when distractor regions were aligned in both versions of the task, whereas function shifts were greatly reduced when the distractor regions were misaligned (**a**). Observers’ susceptibility to the age and gender versions of the composite face illusion correlated closely when distractors were aligned (**b**), but not when they were misaligned (**c**). Error bars = 95% CIs. PSE = point of subjective equality

The distribution of PSEs was analyzed using an ANOVA, with Distractor (female, male) and Configuration (aligned, misaligned) as within-subjects factors. The analysis revealed a significant main effect of Distractor, *F*(1,99) = 132.52, *p* < .001, η_p_^2^ = .57, a nonsignificant main effect of Configuration, *F*(1, 99) = 2.03, *p* = .16, η_p_^2^ = .02, and a significant Distractor × Configuration interaction, *F*(1, 99) = 94.47, *p* < .001, η_p_^2^ = .49. Planned comparisons revealed a significant PSE shift when composites were aligned, *t*(99) = 11.75, *p* < .001. Observers were more likely to judge the target to be male-like in the presence of the male distractor (*M* = .57, *SD* =.08) than in the presence of the female distractor (*M* = .49, *SD* = .08). PSE shifts were significantly reduced in the misaligned compared with the aligned condition, *t*(99) = 9.01, *p* < .001 (see Fig. 3a).

#### Age variant

The distribution of PSEs was analyzed using an ANOVA, with Distractor (child, adult) and Configuration (aligned, misaligned) as within-subjects factors. The analysis revealed a significant main effect of Distractor, *F*(1, 99) = 145.19, *p* < .001, η_p_^2^ = .60, a nonsignificant main effect of Configuration, *F*(1, 99) = .01, *p* = .92; η_p_^2^ < .001, and a significant Distractor × Configuration interaction, *F*(1, 99) = 128.11, *p* < .001, η_p_^2^ = .56. Planned comparisons revealed a significant PSE shift when composites were upright and aligned, *t*(99) = 13.43, *p* < .001. Observers were more likely to judge the target to be child-like in the presence of the child distractor (*M* = .58, *SD* = .12) than in the presence of the adult distractor (*M* = .46, *SD* = .11). PSE shifts were significantly reduced in the misaligned compared with the aligned condition, *t*(99) = 10.52, *p* < .001 (see Fig. 3a).

The PSE shifts seen in the aligned conditions of the gender (*M* = .08, *SD* = .07) and age (*M* = .12, *SD* = .09) tasks correlated significantly (*r* = .43, *p* < .001, *N* = 100, bootstrapped 95% CI [.17, .62]^1^; see Fig. 3b). In contrast, the PSE shifts seen in the misaligned conditions of the gender (*M* = .02, *SD* = .03) and age (*M* = .03, *SD* = .06) tasks did not correlate (*r* = .11, *p* = .29, *N* = 100; see Fig. 3c). The strength of the correlation seen between the aligned variants of the age and gender task was significantly greater than the correlation seen between the misaligned variants (*z* = 2.62, *p* < .01).

## Replication in an online sample

To verify the reliability of the relationship observed, we sought to replicate the correlation in a second sample, tested online. One hundred and thirty-seven typical observers were recruited via Prolific (https://prolific.ac/) and completed both the gender and age variants of our task online. Half the participants completed the age variant first, and half completed the gender variant first. We were unable to model functions for 16 of these observers, leading to a final sample of 121 (*M*_age_ = 28 years, *SD*_age_ = 9.35 years, 61 males). Online versions of the age and gender tasks were programmed in Unity and made available through Unity WebGL (https://unity.com). The stimuli and experimental procedure were identical to that employed in the lab-based study.

### Results and discussion

#### Gender variant

**Fig. 4:**
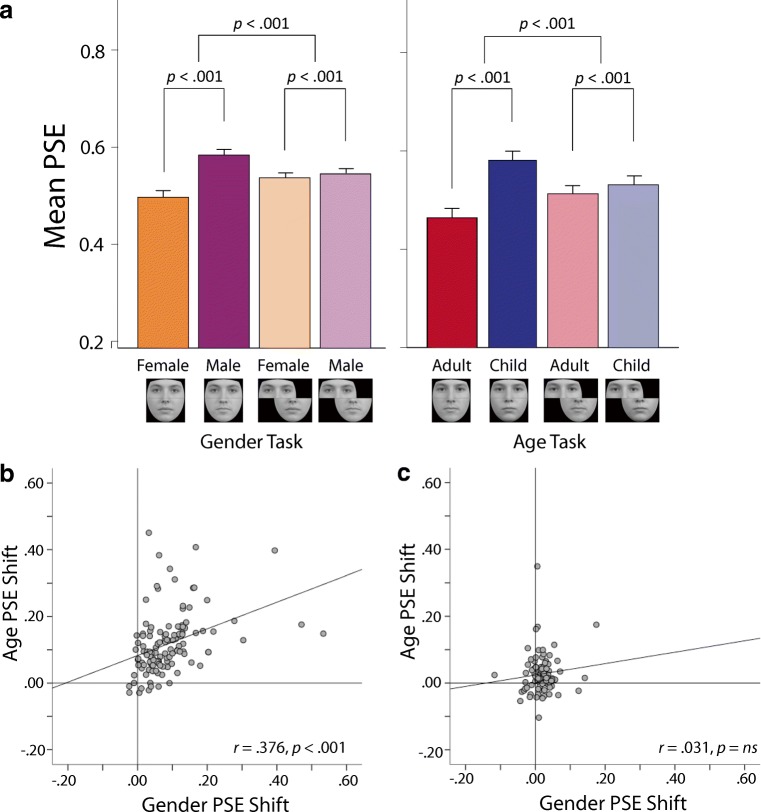
Results from the online sample. Again, substantial function shifts were induced when distractor regions were aligned in both versions of the task, whereas function shifts were greatly reduced when the distractor regions were misaligned (**a**). Observers’ susceptibility to the age and gender versions of the composite face illusion correlated closely when distractors were aligned (**b**), but not when they were misaligned (**c**). Error bars = 95% CIs. PSE = point of subjective equality

The distribution of PSEs was analyzed using an ANOVA, with Distractor (female, male) and Configuration (aligned, misaligned) as within-subjects factors. The analysis revealed a significant main effect of Distractor, *F*(1, 120) = 113.52, *p* < .001, η_p_^2^ = .49, a nonsignificant main effect of Configuration, *F*(1, 120) = .10, *p* = .75; η_p_^2^ < .01, and a significant Distractor × Configuration interaction, *F*(1, 120) = 112.86, *p* < .001, η_p_^2^ = .49. Planned comparisons revealed a significant PSE shift when composites were aligned, *t*(120) = 11.29, *p* < .001. Observers were more likely to judge the target to be male-like in the presence of the male distractor (*M* = .58, *SD* = .07) than in the presence of the female distractor (*M* = .50, *SD* = .08). PSE shifts were significantly reduced in the misaligned compared with the aligned condition, *t*(120) = 10.62, *p* < .001 (see Fig. 4a).

#### Age variant

The distribution of PSEs was analyzed using an ANOVA, with Distractor (child, adult) and Configuration (aligned, misaligned) as within-subjects factors. The analysis revealed a significant main effect of Distractor, *F*(1, 120) = 160.58, *p* < .001, η_p_^2^ = .57, a nonsignificant main effect of Configuration, *F*(1, 120) = .01, *p* = .91, η_p_^2^ < .01, and a significant Distractor × Configuration interaction, *F*(1, 120) = 151.79, *p* < .001, η_p_^2^ = .56. Planned comparisons revealed a significant PSE shift when composites were upright and aligned, *t*(120) = 14.24, *p* < .001. Observers were more likely to judge the target to be child-like in the presence of the child distractor (*M* = .57, *SD* =.09) than in the presence of the adult distractor (*M* = .45, *SD* = .10). PSE shifts were significantly reduced in the misaligned compared with the aligned condition, *t*(120) = 12.32, *p* < .001 (see Fig. 4a).

As in the lab-based study, the PSE shifts seen in the aligned conditions of the gender (*M* = .09, *SD* = .09) and age (*M* = .12, *SD* = .09) tasks correlated significantly (*r* = .38, *p* < .001, *N* = 121, bootstrapped 95% CI [.22, .57]; see Fig. 4b). In contrast, the PSE shifts seen in the misaligned conditions of the gender (*M* = .01, *SD* = .03) and age (*M* = .02, *SD* = .05) tasks did not correlate (*r* = .03, *p* = .74, *N* = 120; see Fig. 4c). The strength of the correlation seen between the aligned variants of the age and gender task was significantly greater than the correlation seen between the misaligned variants (*z* = 2.95, *p* < .01).

## General discussion

The correlation seen between individuals’ susceptibility to the age and gender biases induced by the composite face illusion suggests that these effects are different measures of a common structural binding process. Leading theoretical models hypothesize that structural descriptions, derived early in the face-processing stream, form a common basis for judgements about various facial attributes, such as identity, expression, age, and gender (Bruce & Young, 1986; Haxby et al., 2000). The locus of the structural binding process responsible for the age and gender biases is therefore likely to be early in this processing stream, before the engagement of attribute-specific processing. This conclusion accords with evidence that the composite face illusion modulates the N170, an EEG measure of early face encoding (Jacques & Rossion, 2009).

What kind of structural encoding might generate the composite face illusion? In our day-to-day encounters with faces, we are exposed to naturally occurring sources of feature covariation; male eyes co-occur with male mouths, adult eyes co-occur with adult mouths, and so on. The perceptual models that observers develop in these environments will likely reflect these structural contingencies (Gray, Murphy, Marsh, & Cook, 2017). When presented with contrived composite face arrangements that violate these naturally occurring contingencies, the visual system appears to impose the natural ‘whole-face’ solution that best fits each contrived composite arrangement. As a result of this modeling process, observers’ subjective perception of the target region is biased in the direction defined by the distractor region.

Currently, many authors use matching paradigms to measure the composite face illusion. In these tasks, observers view pairs of target regions, either presented sequentially or simultaneously, and are asked to judge whether they are identical or not (Hole, 1994). Under this approach, individual differences in illusion susceptibility are inferred from observers’ ability (or inability) to discriminate target regions in the presence of distractor regions. While these approaches have proved useful in revealing the normative properties of the illusion, they tell us little about the nature of the illusory biases experienced by observers. For example, where the manifestation of the illusion causes an observer to mistakenly judge identical target regions to be different, it is not clear *how* their two subjective percepts differed. In this vein, we have previously noted that composite effects attributed to the biasing of identity perception may instead reflect the biased perception of expression (Gray et al., 2017). The new psychophysical approach presented here offers a complementary tool with which researchers can study the extent to which a given distractor region biases perception of a target in a particular direction.

### Open practices statement

The data from all experiments are available on the Open Science Framework (10.17605/OSF.IO/T46EC). None of the Experiments described were preregistered.

## Electronic supplementary material

ESM 1 (DOCX 112 kb)

## Footnotes

1 Individuals’ susceptibility to the age and gender illusions also correlated significantly when analyzed using Spearman’s rank correlation. This was true of the lab-based (*r*_s_ = .32, *p* = .001) and online samples (*r*_s_ = .48, *p* = .001).
