# Supplementary material for: Are the facial gender and facial age variants of the composite face illusion products of a common mechanism?
Source: Psychon Bull Rev. 2019 Dec 10;27(1):62–9. doi: 10.3758/s13423-019-01684-9 (PMC7000539; doi:10.3758/s13423-019-01684-9)
Supplement: Supplementary file 1 — (DOCX 112 kb) [file 13423_2019_1684_MOESM1_ESM.docx]

**Are the facial gender and facial age variants of the composite face illusion products of a common mechanism?**

Katie L.H. Gray^1*^, Yvonne Guillemin^2^, Zarus Cenac^2^, Sophie Gibbons^1^, Tim Vestner^3^, Richard Cook^3^

**Establishing test-retest reliability**

Twenty-eight typical observers (*M*_age_ = 23; *SD*_age_ = 5.89; 9 males) completed both versions of our task twice, under controlled lab conditions. Participants had normal or corrected-to-normal vision. For the gender task, we were unable to model psychometric functions for two participants’ data (i.e., there was no systematic relationship between stimulus intensity and their pattern of responding), giving a final sample of 26 for this version of the task.

Testing took place over two hour-long testing sessions. In each session, we sought to model four functions for each observer: aligned child distractor, aligned adult distractor, aligned male distractor, aligned female distractor. Within each session, the tasks were blocked and completed in a counter-balanced order. Each psychometric function was estimated from 140 categorization decisions (7 target levels × 20 presentations). In total, each observer therefore completed 1120 trials (2 facial attributes × 2 levels of distractor × 140 trials per function × 2 testing sessions).

*Results and discussion*

*Gender variant.* Observers were more likely to judge the target to be male-like in the presence of the male distractor than in the presence of the female distractor in both the first session (Male distractor: [*M* = .58; *SD* =.08]; Female distractor: [*M* = .49; *SD* = .06]; [*t*(25) = 6.59, *p* < .001], and the second (Male distractor: [*M* = .62; *SD* =.12]; Female distractor: [*M* = .53; *SD* =.10]; [*t*(25) = 6.44, *p* < .001]). The test-retest reliability of this version of the task was high [*r* = .76, *p* < .001] (Figure S1a).

*Age variant*. Observers were more likely to judge the target to be child-like in the presence of the child distractor than in the presence of the adult distractor in both the first session (Child distractor: [*M* = .64; *SD* =.17]; Adult distractor: [*M* = .49; *SD* = .14]; [*t*(27) = 5.53, *p* < .001]), and the second (Child distractor: [*M* = .65; *SD* =.08]; Adult distractor: [*M* = .51; *SD* =.10]; [*t*(27) = 6.28, *p* < .001]). The test-retest reliability of this version of the task was also good [*r* = .60, *p* = .001] (Figure S1b).


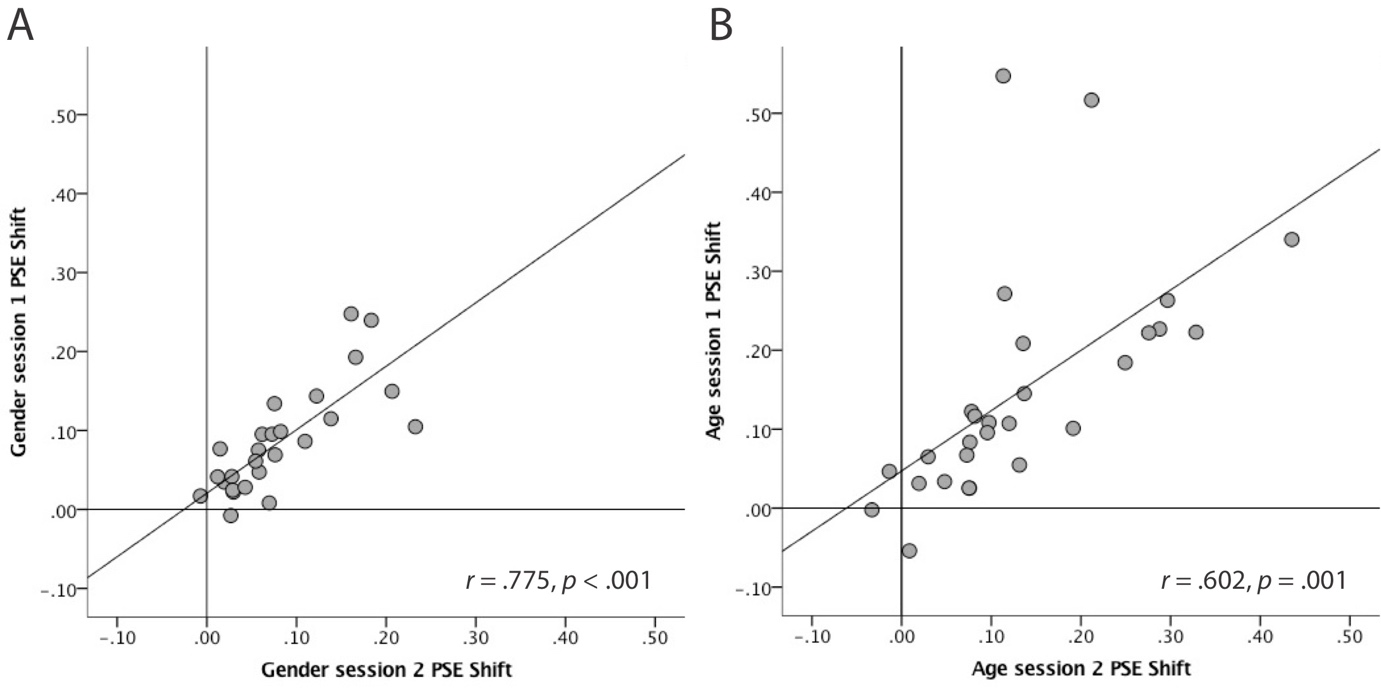


Figure S1. Scatterplots of the test-retest data (session 1 plotted against session 2) for the Gender task (A) and Age task (B).
